# Prevalence of asymptomatic malaria infection and use of different malaria control measures among primary school children in Morogoro Municipality, Tanzania

**DOI:** 10.1186/s12936-015-1009-4

**Published:** 2015-12-02

**Authors:** Baraka J. Nzobo, Billy E. Ngasala, Charles M. Kihamia

**Affiliations:** Department of Parasitology and Medical Entomology, Muhimbili University of Health and Allied Sciences, PO Box 65011, Dar es Salaam, Tanzania

**Keywords:** Asymptomatic, Malaria, School children, ITN use, ACT, Municipality

## Abstract

**Background:**

Malaria is a public health problem in Tanzania affecting all age groups. It is known that school children are the age group most commonly infected with malaria parasites. Their infections are usually asymptomatic, go unnoticed and thus never get treated, result in anaemia, reduced ability to concentrate and learn in school and if fallen sick may lead to school absenteeism. Effective malaria control requires frequent evaluation of effectiveness of different malaria interventions.

**Methods:**

A cross-sectional study design involving 317 out of 350 school children aged 6–13 years from five primary schools within municipality was conducted. Multistage cluster sampling and simple random sampling methods were used to obtain primary school and study participants, respectively. Finger-prick blood samples were collected for *Plasmodium* parasite detection by malaria rapid diagnostic test (mRDT) and haemoglobin level assessment by Easy Touch^®^ GHb system machine. A questionnaire was administered to assess use of insecticide-treated nets (ITNs) and anti-malarial drugs.

**Results:**

The prevalence of asymptomatic malaria was 5.4 % (95 % CI 3.3–8.6 %) and anaemia was 10.1 % (95 % CI 7.2–13.9 %). School children aged 6–9 years were more affected by malaria than those aged 10–13 years. The proportion of ITNs used was 90.6 % (95 % CI 86.3–93.9 %) while that of artemisinin combination therapy (ACT) was 71.9 % (95 % CI 66.2–77.1 %).

**Conclusion:**

Findings show existence of asymptomatic malaria and walking anaemia among primary school children in Morogoro municipality. The majority of school children reported use of ITNs and ACT for malaria control. These findings provide a rationale for using schools and school children to assess effectiveness of malaria interventions.

## Background

Malaria infection is caused by five species of parasite of the genus *Plasmodium* that affect humans: *P. falciparum, P. vivax, P. ovale, P. malariae,* and *P. knowlesi* [1]. In malaria-endemic countries, many *P. falciparum* infections are asymptomatic. The asymptomatic carriers do not seek treatment for their infection and, therefore, add up to a reservoir of parasites available for transmission by *Anopheles* mosquitoes [2].

Malaria is a public health problem in Tanzania and the major cause of morbidity and mortality, accounting for about 30 % of all hospital admissions and 15 % of all hospital deaths [3, 4]. Asymptomatic malaria infection in healthy Tanzanians was reported in 1986 when 8.1 % were found to have asymptomatic malaria parasitaemia; their prevalence was highest among young age groups and lowest in older age [5]. Prevalence of asymptomatic malaria infection among primary school children was reported as 14.3 % in northwestern Tanzania [6]. It is reported that school-age children are the age group most commonly infected with malaria parasites. Their infections are usually asymptomatic, go unnoticed and thus never get treated [7], resulting in anaemia, reduced ability to concentrate and learn in school, and if sick may lead to school absenteeism [8].

The use of artemisinin-based combination therapy (ACT) in combination with insecticide-treated nets (ITNs), long-lasting, insecticidal nets (LLINs), and indoor residual spraying (IRS) is perhaps the most aggressive methods for reducing the malaria burden in endemic regions [9]. An observational study in Tanzania shows that the introduction of ACT at fixed health facilities only modestly reduced asexual parasitaemia prevalence. ACT is effective for treatment of uncomplicated malaria and should have substantial public health impact on morbidity and mortality, but is unlikely to reduce malaria transmission substantially in much of sub-Saharan Africa where individuals are rapidly re-infected [10, 11].

Studies revealed that ITNs used for protection against mosquito bites have proven to be a practical, highly effective and cost-effective intervention against malaria. A decline in malaria in sub-Saharan Africa is attributed to malaria control measures, predominantly to the use of ITNs, IRS and ACT, which have been implemented on a wide scale [12].

Malaria control efforts in Africa have been intensified in young children; this has resulted in exposure-dependent immunity and increases the risk of malaria transmission to school-aged children and above. But little is known about the burden of malaria in African school children and evidence suggests that malaria causes up to 50 % of all deaths in this age group [13].

## Methods

### Study area

This study was conducted in Morogoro municipality, which is located at altitude 500–600 m above sea level and between longitude 37º–39ºE and latitude 6º–5ºS. The municipality consists of the town of Morogoro, one of the oldest towns in Tanzania, located about 223 km southeast of the capital city Dodoma, and 195 km west from the commercial city of Dar es Salaam, and has a population of 315,866. It is the capital of the Morogoro region and enjoys a mixture of warm and cool temperatures ranging between 27 and 33.7 °C in the dry/warm season and 14.2 and 21.7 °C in cold/wet season.

### Study population

The study was conducted in 5 out of 60 public primary schools within the municipality. The unit of analysis was primary school children both male and female aged 6–13 years randomly selected within the five primary schools.

### Sampling techniques

Multistage cluster sampling method was used to obtain the required primary schools and their respective classes. First stage: all 60 public primary schools in the municipality were divided into five clusters, namely (1) schools around mount Uluguru; (2) schools around swampy areas; (3) schools located in the town centre; (4) schools in peri-urban areas; and, (5) schools in squatter areas. From the five clusters, five primary schools were randomly selected, namely: Mlimani, Jitegemee, Uhuru, Kingolwira, and Mafisa A. The second stage was to select one representative class (from standard 1–6) for each selected school. Then simple random sampling technique was used to recruit 70 children in each school who were available on the sampling day. The selected children were given a written informed consent form for their parents or guardians to read and sign if they agreed their children could participate in the study. For those parents who could not read, the children were instructed to read for them and also to tell their parents to finger print in the signature area.

### Data collection techniques

A structured and pre-tested questionnaire was used to collect information on demographic characteristics, knowledge about the transmission and prevention of malaria, ownership and utilization of ITNs, and use of ACT of all 317 out of 350 eligible participants. To obtain an asymptomatic individual, a thorough medical history was taken of all 317 school children by asking if a child had the following features: history of fever within the last 24 h, headache and generalized joint pain plus measuring axillary body temperature <37.5 °C by thermometer. Fortunately all 317 school children showed no clinical signs and symptoms of malaria. The interviews were conducted in Swahili in a separate classroom from that used for haemoglobin and malaria examination. A finger-prick blood sample was collected after cleaning the finger surface using a sterile cotton swab saturated with 70 % isopropyl alcohol.

### Parasitological examination

Presence of malaria parasites among asymptomatic school children was ascertained during the study survey by using multi-species malaria rapid diagnostic test (*P. falciparum* histidine-rich protein II (PfHRP-II) and other *Plasmodium* species (Pan, pLDH) (*P. vivax, P*. *malariae* or *P. ovale*) type SD 05FK60.

### Haemoglobin measurements

Haemoglobin level was measured at the field using portable Easy Touch^®^ GHb system machine by examining peripheral blood from finger pricks. Definition of anaemia was based on WHO criteria definition where haemoglobin level <7 g/dl is regarded as severe anaemia, between 7 and 9.9 g/dl is moderate anaemia, between 10 and 10.9 g/dl is mild, while haemoglobin level of ≥11 g/dl is regarded as normal.

### Quality control

Microscopy quality control was conducted whereby thick blood films was stained at Morogoro Regional Referral Hospital after the survey, using 10 % Giemsa stain, and examined after completion of fieldwork by an experienced laboratory technician in the Hospital. A second reading was carried out by a highly experienced technician at Muhimbili University of Health and Allied Sciences-Parasitology Laboratory and results were compared. All technicians were blinded for malaria rapid diagnostic test (mRDT) results.

### Data management and analysis plan

Data entry, storage and analysis were done by the principal investigator (PI) using SPSS version 15 software. The double entry was performed for raw data to minimize possible errors during data entry. Frequencies and cross tabulation were calculated to obtain proportions and Chi squared tests for each of the study variables. Chi square test and logistic regression analyses were done to obtain the association and relationship between independent and dependent variables. P value of <0.05 at 95 % CI was used to test the statistical significances between the dependent and independent variables. Epi Info version 3.5.4 was used to calculate the 95 % CI of proportions. Results are presented in the Tables and Figures.

### Ethical considerations

Ethical clearance was sought from Muhimbili University of Health and Allied Sciences before commencing the study. The Institution Review Board (IRB) reviewed the proposal of this research study and advised accordingly. After receiving research ethical clearance, other permission was requested from Executive Municipal Director (EMD) through Municipal Education Officer to allow the PI to conduct the research study on their school children. Informed consent was requested from each primary school head teacher after receiving a permission letter from Municipal Education Officer, and finally a written informed consent form was given to the selected school children. The selected school children were requested to take the written informed consent to their parents or guardians and request them to read and sign if they allowed their children to participate in the study. All selected children were instructed to bring back the signed informed consent forms. Parents/guardians who did not want their children to participate in the study were free to refuse participation. If a parent or guardian chose not to allow their children to participate in the survey, the child’s name was removed from the participant list. During data collection procedures, participants who were diagnosed with anaemia and any other health-related problems were referred to the nearest health facility for further medical management.

## Results

A total of 317 out of 350 school children were recruited in the study after receiving the signed informed consent from their parents, but parents of 33 out of 350 primary school children refused to allow them to participate in this study. The mean age was 9.85 years (standard error of the mean (SE) = 0.106, standard deviation (SD) = 1.891).

### Prevalence of asymptomatic malaria

The overall prevalence of asymptomatic malaria on RDT was 5.4 % (95 % CI 3.3–8.6 %) and 7.6 % (95 % CI 5.0–11.2 %) were positive to microscopy as quality control. Females were more affected with asymptomatic malaria than males (OR = 1.76). School children aged 6–9 years were slightly more affected with malaria than those aged 10–13 years (OR = 0.93). In this study, it was shown that primary schools located in peri-urban areas (Kingolwira primary school) had more children with asymptomatic malaria (13 %) than those located in other parts of the municipality and their observed differences were statistically significant (P = 0.012) as shown in Table 1.

**Table 1:** Prevalence of asymptomatic malaria with sex, age groups and school clusters

Covariates	Category	N	Positive	P value
Sex	Male	132	5 (3.8 %)	0.293
	Female	185	12 (6.5 %)	
Age group (years)	6–9	137	8 (5.8 %)	0.742
	10–13	180	9 (5.0 %)	

Clusters	Schools	N	Positive	P value
Swampy areas	Jitegemee	60	2 (3.3 %)	0.012
Peri-urban	Kingolwira	68	9 (13.2 %)	
Squatter areas	Mafisa A	69	4 (5.8 %)	
Mount Uluguru	Mlimani	54	2 (3.7 %)	
Town centre	Uhuru	66	0 (0 %)	

The association between asymptomatic malaria parasitaemia and demographic characteristics (sex and age groups) of primary school children was done using Chi square test (P < 0.05 at 95 % CI). From this study, being a male child seemed to be a protective factor for malaria infection (OR = 0.568, i.e., OR < 1). Females were slightly more affected with malaria parasites although it was not statistically significant (P = 0.293). Age group had no association with malaria infection (OR = 1.178, i.e., OR = 1) and not statistically significant (P = 0.742). Logistic regression was performed; no variables were statistically significantly associated with asymptomatic malaria infection.

### Prevalence of anaemia

The overall point prevalence of anaemia (haemoglobin level <11 g/dl) was 10.1 % (95 % CI 7.1–14.1 %). The mean haemoglobin level was 13.4 g/dl (95 % CI 13.181–13.677 %) with minimum level of 5.0 g/dl and maximum level of 21.5 g/dl.

**Fig. 1:**
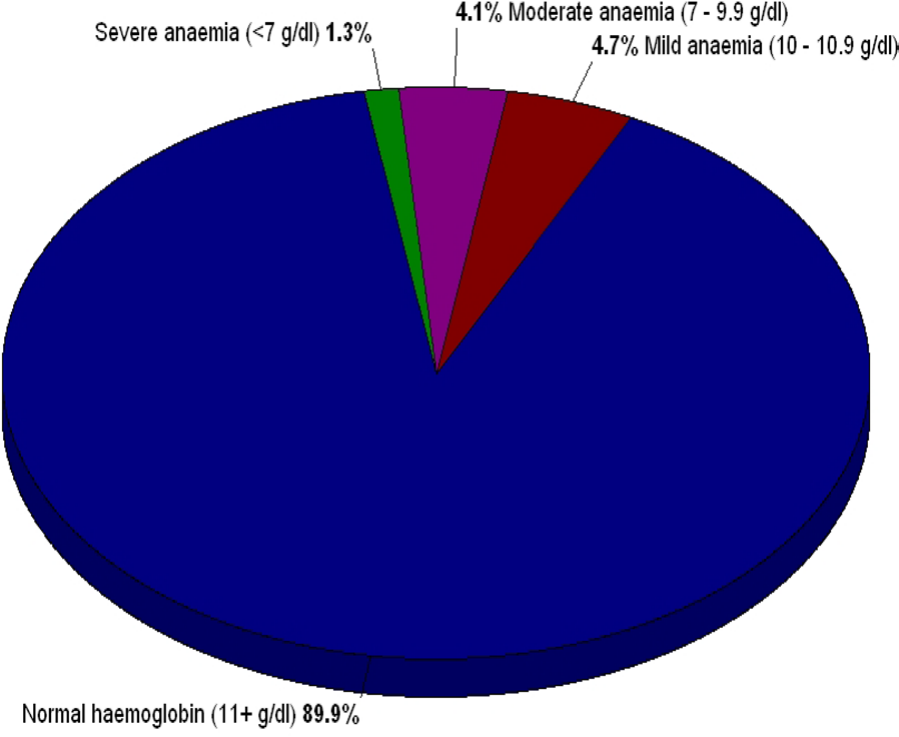
Haemoglobin level distribution among primary school children (N = 317). Haemoglobin level was measured in the field using portable Easy Touch^®^ GHb system machine by examining peripheral blood from finger pricks. It was categorized as follows: haemoglobin level <7 g/dl, severe anaemia, haemoglobin level between 7 and 9.9 g/dl, moderate anaemia, haemoglobin level between 10 and 10.9 g/dl, mild anaemia, haemoglobin level of (11 + g/dl) or ≥11 g/dl, normal haemoglobin level

Among 317 school children, four (1.3 %) had severe anaemia (haemoglobin level <7 g/dl), 13 (4.1 %) had moderate anaemia (haemoglobin level 7–9.9 g/dl) and 15 (4.7 %) had mild anaemia (haemoglobin level 10–10.9 g/dl) as shown in Fig. 1. However, anaemia varies with sex and age groups; 12.4 % of anaemic cases were from age group 6–9 years, while 8.3 % were in age group 10–13 years (P = 0.233). Females were more anaemic than males with proportions 11.4 and 8.3 %, respectively (P-value = 0.379). However, all who had been diagnosed malaria positive with mRDT and microscopy had normal haemoglobin, while all anaemic cases (10.1 %) were malaria negative. Prevalence of anaemia was further analysed with the past history of malaria infection. Results showed that 75 % of anaemic cases had previously suffered from malaria while 25 % of anaemic cases had not suffered from malaria, the observed difference was statistically significance (χ^2^ = 6.582, P = 0.01).

### ITN and ACT use

In the study participants, a total of 254 (80.1 %) (95 % CI 75.4, 84.4 %) school children reported to have an ITN and to sleep under it, but 63 (19.9 %) school children did not have an ITN (N = 317). Those who reported to have ITNs in their home (N = 254), were asked about their use: 207 (81.5 %) (95 % CI 76.2, 86.1 %) sleep under an ITN every night, 21 (8.3 %) sleep under an ITN when fallen sick, and 26 (10.2 %) sleep under an ITN a few nights.

To minimize recall bias, children were asked about the use of an ITN the night before the interview: 230 (90.6 %) (95 % CI 86.3, 93.9 %) of school children reported sleeping under an ITN the night before the interview and 24 (9.4 %) reported not sleeping under an ITN the night before the interview. Males were observed to use ITNs more than females; their proportions were 92.4 and 89.3 %, respectively (P = 0.403). Moreover, school children in age group ten to 13 years were observed to use ITNs more (91.7 %) compared to the age group 6–9 years (P = 0.461) as shown in Table 2. There was no statistical significance in the observed difference for the use of ITNs by age groups and sex.

**Table 2:** Proportion of insecticide-treated net use the previous night by age and sex (N = 254)

Demographic variables	Category	Insecticide-treated net (ITN) use		P value
		Yes	No	
Sex	Male	97 (92.4 %)	8 (7.6 %)	0.403
	Female	133 (89.3 %)	16 (10.7 %)	
Age groups (years)	6–9	97 (89.0 %)	12 (11.0 %)	0.461
	10–13	133 (91.7 %)	12 (8.3 %)	

**Fig. 2:**
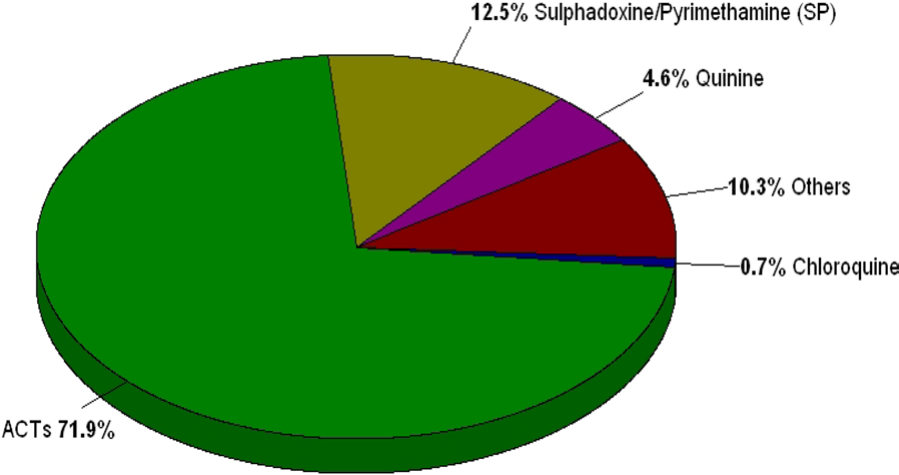
Distribution of anti-malarial drugs used among primary school children (N = 281). Common anti-malarial drugs reported to be used in this study by the primary school children were as follows: artemisinin based combination therapy (ACT), sulfadoxine–pyrimethamine (SP), quinine (both tablets and injections), chloroquine tablets, and others (medications such as paracetamol and amoxicillin)

Primary school children were asked if they remembered suffering from malaria. Responses enabled the question about use of anti-malarial medicines. A total of 281 (88.6 %) of primary school children remembered suffering from malaria at different times of the year; 36 (11.4 %) did not remember if they had suffered from malaria (N = 317). From 281 primary school children who had suffered from malaria, 202 (71.9 %) (95 % CI 66.2–77.1 %) used ACT, 35 (12.5 %) used sulfadoxine–pyrimethamine, 13 (4.6 %) used quinine, two (0.7 %) used chloroquine and 29 (10.3 %) used other medications such as paracetamol and amoxicillin (Fig. 2).

## Discussion

This study aimed to determine the prevalence of asymptomatic malaria infection and anaemia and several malarial control measures among primary school-aged children in Morogoro municipality. Despite the decline of malaria infection in Tanzania, the disease still remains a public health problem affecting all age groups in the community, including primary school children. Malaria infections in primary school children are mostly asymptomatic, and therefore form a group of individuals called asymptomatic carriers. These asymptomatic carriers act as reservoir of malaria parasites in the community readily available for transmission by female *Anopheles* mosquitoes. Usually malaria asymptomatic carriers are not detected early and treated, thus these infections may result in anaemia, reduced ability to concentrate and learn in school, and if sick may lead to school absenteeism.

### Prevalence of asymptomatic malaria among primary school children

These findings showed low prevalence of asymptomatic malaria (5.4 and 7.6 %) on mRDT and microscopy, respectively, among school children in Morogoro Municipality. These findings are similar to other reports in urban cities of sub-Saharan Africa: Brazzaville 4.0 % and 3.6–7.5 % in Dakar [14]. A national school malaria survey in Kenya reported similar prevalence of 4.3 % as those of urban cities of sub-Saharan Africa [15]. However, results from this study were different from a malaria national survey in Côte d’Ivore which reported highest prevalence of asymptomatic malaria (63.3 %) in school children aged 5–16 years [16]. The observed difference of malaria detection between mRDT type 05FK60 (diagnostic) and microscopy (quality control) in this study was due to the fact that sensitivity of SD 05FK60 improved with increasing parasitaemia level >50 parasites/µl of blood [17, 18].

This study revealed that primary school children aged 6–9 years were more affected with asymptomatic malaria than the other age group. Female school children were found to be more affected than male school children. Similar finding have been documented in a study done in Nyamatongo Ward, northwestern Tanzania [6]. This study also revealed the highest reported proportion of school absenteeism (74.7 %) for those children who reported suffering from malaria in the past few months. School children with asymptomatic malaria will often, at any time, change to symptomatic (febrile) and hence create the possibility of school absenteeism. This observation was similarly reported on the Tanzania demographic health survey [19], malaria indicator survey [4] and in a study of asymptomatic malaria among school children in Yemen [20].

Demographic characteristics, such as age and sex, were analysed to find the association with asymptomatic parasitaemia. Males were less affected with asymptomatic malaria than females (OR < 1) although it was statistically not significant (P = 0.293). Age groups seemed to have no association with asymptomatic malaria (OR = 1) but statistically not significant (P = 0.742). Binary logistic regression was performed to control for any possible confounder such as ITN use in the association. Age group 6–9 years had 1.2 times more chance (OR = 1.231, P = 0.68) to be asymptomatic malaria carrier than age group 10–13 years. Males continue to be less affected with asymptomatic malaria than females (OR < 1, P = 0.295). Similar findings were obtained in Nyamatongo Ward, northwestern Tanzania which reported that males were less affected with asymptomatic malaria [6]. This is probably because male school children tend to sleep earlier (reduced exposure time to mosquito bites) at night than females who delay (increased exposure time to mosquito bites). In African cultures, female children had responsibilities to assist their mothers in several sociocultural activities once they came back from school until the late hours of a day, and hence the chances of being bitten by mosquitoes is higher than in males.

### Prevalence of anaemia among primary school children

Anaemia is a public health problem affecting people in both developed and developing countries with bad consequences on human health as well as social and economic development. It is a critical health concern because of its effects on growth, energy levels and immune mechanism. The overall prevalence of anaemia obtained from this study was 10.1 % (95 % CI 7.2, 13.9 %) and was statistically significant (P = 0.01) to current malaria infection history (1–6 months prior). This prevalence was similar to the one reported from Kisarawe, Tanzania by Manangwa (2012, pers comm) but was low compared to the prevalence among school children in Tanga region, Tanzania, which was 79.6 % [21].

However, there was a contrasting result in this study: the observed anaemia was not statistically significant with the prevalence of asymptomatic malaria obtained (RR = 1, P = 0.156). This means that malaria is not the only cause of anaemia among school children, other causes such as nutritional anaemia, hookworm and schistosomiasis infections should be considered; a similar conclusion was reported in Tanzania’s demographic health survey [19]. In this study, females had been observed to be more anaemic than males although their observed difference was not statistically significant. Similar findings were reported in Bangalore, south India in school children aged 5–15 years, although their observed difference was statistically significant [22]. Difference in statistical significance can be explained on the basis of large sample size used in Bangalore compared to the small sample size in this study.

### ITNs and ACT use

In this study, it was revealed that 80.1 % (95 % CI 75.3, 84.4 %) of primary school children reported having ITNs in their bedrooms; this proportion is low compared to the general Morogoro region proportion of 91 % ITN coverage. The reason for observed difference of ITN coverage was clearly explained in Tanzania’s HIV/AIDS malaria indicator survey 2011–2012, that rural households are more likely than urban households to own an ITN (92 and 87 %, respectively). This study has the highest ITN coverage in primary school children compared to a study in Kenya which reported 44.2 % (95 % CI 42.7–45.6 %) ITN use [15].

Frequency of use of ITNs differs among those who reported to have ITNs in their bedrooms. A majority, 81.5 % (95 % CI 76.2, 86.1 %) reported using an ITN every night and the remaining few reported using an ITN when fallen sick or on a few nights. A good indicator of ITN use was in those who reported sleeping under an ITN the night before data collection day. The proportion of primary school children who slept under an ITN the night before data collection was 90.6 % (95 % CI 86.3–93.9 %), thus meeting the Roll Back Malaria/WHO artnership target that ITN coverage and use in the target community should be ≥ 80 % [23]. This proportion is higher than that of 42.1 % (95 % CI 40.9–42.8 %) which was reported in Kenya [15] and higher than 69.6 % overall use of ITNs for Tanzanian children aged 5–14 years [4]. Again, this highest use of ITNs observed in this study was different from observations made in Uganda, Ghana and the Gambia, that children between ages 5–14 years had significantly lower usage of ITNs [24].

The proportion of ACT use observed in this study was 71.9 % (95 % CI 66.2–77.1 %) among primary school children who reported having suffered from malaria infection. This proportion is low for the Roll Back Malaria/WHO partnership target that 80 % of people confirmed to have uncomplicated malaria be treated with ACT within 24 h [23]. A similar observation of the lowest use of ACT as per WHO target was reported in a study done in Kenya [25]. Although malaria reduction is achieved through combined strategies, ACT has played an important role in reducing the malaria burden in Zanzibar, Rwanda and São Tomé and Príncipe. It was predicted that, when artemether–lumefantrine (ALU) was used as mass drug administration to asymptomatic carriers in the community screening campaigns, it may significantly reduce malaria transmission [10, 28]. The higher proportion of ACT use than other monotherapy anti-malarial drugs in this study could be due to the increased belief among Tanzanians about these drugs. For example, one study in Tanzania explained that ALU had >90 % cure rates and well-tolerated treatment in children with uncomplicated malaria [26]. This could be one of the reasons for increased use of ACT among febrile primary school children.

Another finding from this study showed that school children who currently suffered from malaria infection were 1.4 times more likely to use ACT than those who suffered malaria infection more than 2 months previously. Their relationship to ACT use was statistically not significant because some individuals still use sulfadoxine–pyrimethamine (12.5 %) and monotherapy anti-malarial drugs, such as quinine (4.6 %) and chloroquine (0.7 %). Since *P. falciparum* has developed resistance to commonly used anti-malarials, such as chloroquine and sulfadoxine–pyrimethamine, continued use of them will lead to major challenges to malaria control strategies. Recommendations to use ACT in treatment of uncomplicated malaria should be adhered to [27].

## Conclusion

The significantly low prevalence of asymptomatic malaria in this study can be due to the reported high proportion of ITN and ACT use among primary school children in Morogoro municipality. Even if there was no statistical significance, school children age 6–9 years and females were the most affected groups with asymptomatic malaria than other groups (i.e. males and children ages 10–13 years). The observed prevalence of anaemia was not associated with the diagnosed asymptomatic cases (all asymptomatic malaria positive cases in this study had normal haemoglobin level). This means that malaria is not the only cause of anaemia; other factors such as hookworm, schistosomiasis and nutritional anaemia should be considered. Severe anaemia cases were also among the findings of this study even though its prevalence was low; having severe anaemia is life threatening, therefore, further study should be carried out to find the predictors of anaemia among primary school children in Morogoro municipality. School malaria surveys, if well arranged, can be complimentary to current malaria indicator surveys in Tanzania, which survey children under 5 years and pregnant women.

## Abbreviations

ACT: artemisinin-based combination therapy
ITNs: insecticide-treated nets
ALU: artemether lumefantrine
SP: sulfadoxine–pyrimethamine
RDT: rapid diagnostic test
LLINs: long-lasting insecticidal nets
IRS: indoor residual spray
